# Veterinary Drug Residues in Food Chains: Sources, Exposure Pathways, Health Impacts, Mitigation, and Safety Assurance

**DOI:** 10.3390/foods15050840

**Published:** 2026-03-03

**Authors:** Yiting Wang, Jiacan Wang, Linwei Zhang, Shiyun Han, Xiaoming Pan, Hao Wen, Hongfei Yang, Xu Wang, Dapeng Peng

**Affiliations:** 1 Zhejiang Provincial Engineering Research Center of Animal Biological Products, Center for Veterinary Sciences, Zhejiang University, Hangzhou 310058, China; hanshiyun2022@163.com; 2 National Reference Laboratory of Veterinary Drug Residues (HZAU) and MOA Key Laboratory for Detection of Veterinary Drug Residues, Huazhong Agricultural University, Wuhan 430070, China; 15137603562@163.com (Y.W.); wangjiacan0805@163.com (J.W.); zhanglinwei9910@163.com (L.Z.); pxiaoming2596@163.com (X.P.); w793653717@163.com (H.W.); tiep159902@163.com (H.Y.); wangxu@mail.hzau.edu.cn (X.W.)

**Keywords:** veterinary drug residues, food safety, antibiotic resistance, mitigation strategies, alternatives to antibiotics, detection technology

## Abstract

The residues of veterinary drugs in the food chain are a global concern for food safety, including questions about the origin of these residues, exposure pathways, health impacts, methods for their dissolution, and accurate monitoring methods. In recent years, numerous professional studies have addressed the above concerns from various perspectives. However, these studies are relatively scattered and cannot provide a systematic and comprehensive understanding of recent developments. In this systematic review, we aim to provide a comprehensive synthesis of the current state of knowledge concerning the residues of veterinary drugs in the food chain through critical examination of their origins, exposure pathways, and associated health/environmental hazards. Investigating creative mitigation techniques to lower such residues in food products is given special attention. In summary, this research proposes a paradigm that balances the development of animal production with strict food safety governance to address productivity, consumer health, and international standards.

## 1. Introduction

**Figure 1:**
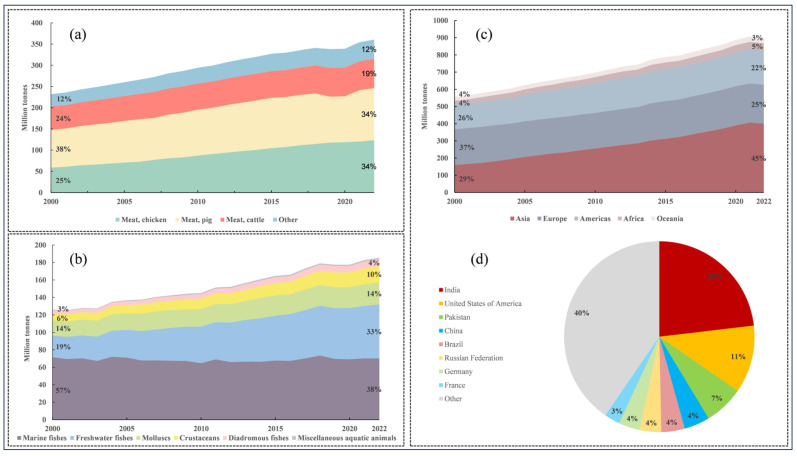
World Food and Agriculture Production Statistics. (**a**) World production of meat, main items; (**b**) world capture fisheries and aquaculture production by species group; (**c**) world production of bovine milk by region; (**d**) world production of bovine milk by main producers (2022) [1].

Animal-derived foods are rich in high-quality protein, peptides, amino acids, lipids, vitamins, and minerals, playing a vital role in human health. According to data from the FAO Statistical Pocketbook—World Food and Agriculture 2024 [1], global demand for animal-derived foods has continued to grow over the past two decades. The structure and trends of global meat production (Figure 1a) reveal that total meat output rose from approximately 230 million tons in 2000 to 360 million tons in 2020, achieving an average annual growth rate of 2.2%. By category, poultry (chicken) increased its share from 25% in 2000 to 34% in 2020, tying with pork as the largest meat category. Global fisheries production (including capture and aquaculture) (Figure 1b) increased from approximately 126 million tons in 2000 to 185 million tons in 2020, with an average annual growth rate of 1.9%. Aquaculture now contributes more than wild capture, accounting for 54% of total global fisheries production. Global milk production (Figure 1c) increased from approximately 556 million tons in 2000 to 897 million tons in 2020, with an average annual growth rate of 2.4%. In 2022, the top five milk-producing countries were India (23%), the United States (11%), Pakistan (7%), China (4%), and Brazil (4%), collectively contributing nearly half of global milk output [1]. To meet the growing demand for animal-derived foods, the development of intensive animal production systems has exacerbated antibiotic misuse. Statistics indicate that antimicrobial drug usage in the animal sector accounted for 73% of global antimicrobial consumption in 2017. Mulchandani et al. projected that global antimicrobial usage is expected to increase by 8% by 2030, reaching 107,472 metric tons [2].

In response to this global challenge, the “One Health” concept has been developed. This approach, supported by the Quadripartite Alliance comprising the Food and Agriculture Organization (FAO), the World Health Organization (WHO), the World Organisation for Animal Health (WOAH, founded as OIE), and the United Nations Environment Programme (UNEP), aims to address issues through multi-level collaboration at local, national, and international levels to safeguard the health and well-being of humans, animals, and the environment [3]. Proposed by the European Commission and its Directorate-General for Health and Food Safety (DG SANTE), Regulation (EU) 2017/625 of the European Parliament and of the Council, known as the Official Controls Regulation, serves as a pivotal legal instrument for implementing the “One Health” principle within the EU and constitutes the cornerstone of EU food and feed production legislation [4]. This regulation establishes the overarching legal obligation for Member States to conduct official controls along the agri-food chain and explicitly incorporates the monitoring of veterinary drug residues (VDRs) within its scope [4]. Commission Delegated Regulation (EU) 2022/1644 and Commission Implementing Regulation (EU) 2022/1646 supplement Regulation (EU) 2017/625 [4,5,6]. Specifically, Regulation (EU) 2022/1646 governs the official controls of pharmacologically active substances and their residues [6]. Regulation (EU) 2022/1644 establishes harmonized rules to ensure a consistent approach in the official controls of residues of pharmacologically active substances, including in imported animals and animal-derived products [5]. It mandates that exporting third countries submit risk-based control plans, providing guarantees at least equivalent to those applicable within the EU and complying with specified minimum sampling frequency requirements [5,6]. In the United States, the Food Safety and Inspection Service (FSIS) of the Department of Agriculture is responsible for the final residue monitoring of meat, poultry, and egg products [7]. Its National Residue Program is executed through systematic sampling and testing at the processing stage to ensure market compliance with safety standards. The Food and Drug Administration (FDA) regulates animal feed and oversees the pre-market approval of all veterinary drugs, including antimicrobials. Its Guidance for Industry #213 (GFI #213) on the Veterinary Feed Directive is a key policy for reducing antimicrobial use. It guides veterinary drug manufacturers to voluntarily withdraw antimicrobials used for growth promotion from the feed market and transition them to prescription-only status [8]. China employs a system centered around the annual “Monitoring Plan for VDRs in Livestock and Poultry Products” issued by the Ministry of Agriculture and Rural Affairs. This plan delineates nationwide sampling tasks, priority products, and target substances. Provincial-level departments of agriculture and rural affairs are responsible for formulating and implementing specific monitoring plans within their respective jurisdictions [9]. Concurrently, with increasingly close international connections, the trend of the “globalization of the dining table” has become more pronounced. In response, major economies have established distinctive food safety databases and information platforms. The European Commission developed the Rapid Alert System for Food and Feed (RASFF), which functions as a monitoring and reporting tool for food safety risks and consumer protection within the EU and the European Economic Area [10]. The United States relies on databases such as the Import Refusal Report (IRR) and the Inspection Classification Database (ICD) to conduct stringent risk screening and categorical management of incoming food products [11,12]. In China, the State Administration for Market Regulation (SAMR) regularly publishes the results of its sampling inspections for imported, exported, and domestically marketed food products on its official government website [13].

However, due to farmers’ lack of scientific knowledge and excessive pursuit of economic gains, the misuse of veterinary drugs is widespread in the livestock industry. Sources of VDRs primarily include feed contamination, Irrational use of drugs, too Short a Withdrawal Period, and environmental pollution. Humans may be exposed to these residues through dietary intake, drinking water consumption, and occupational exposure. Long-term exposure to trace antibiotics in food can lead to increased antimicrobial resistance (AMR), gut microbiota disruption, allergic reactions, and organ toxicity risks. It may even cause long-term health issues such as carcinogenicity, teratogenicity, and mutagenicity. Therefore, strategies to mitigate VDRs in the food chain include the rational use of veterinary drugs in farming, such as adhering to recommended dosages, withdrawal periods, and using drugs only when medically necessary. Additionally, enhanced education and oversight for veterinarians, farmers, and food processors are indispensable. In recent years, heightened awareness of antibiotic misuse has spurred the development of alternatives such as probiotics, phages, organic acids, antimicrobial peptides, botanical extracts, nanoparticles, and antibodies (Abs), offering new approaches to prevent antibiotic overuse. Simultaneously, leveraging advanced analytical techniques—such as Time-Resolved Fluoro Immunoassay (TRFLA) and nanomaterial-based biosensors—to enhance monitoring of these products is crucial for detecting trace residues in complex food matrices.

This systematic review provides a comprehensive summary of the current state of knowledge regarding VDRs in the food chain through an in-depth analysis of their origins, exposure pathways, and associated health/environmental hazards. Particular emphasis is placed on exploring innovative mitigation strategies to reduce such residues in food, aiming to safeguard public health and promote the ecological sustainability of livestock production systems.

## 2. The Sources of Veterinary Drug Residues in Food Chains

**Figure 2:**
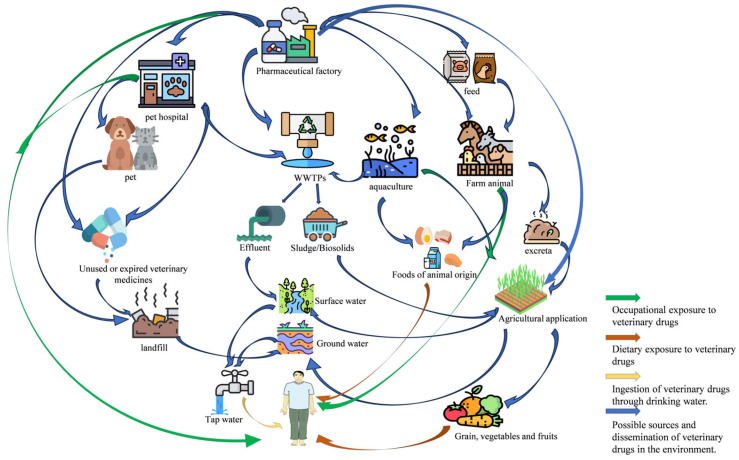
Sources of veterinary drug residues in the food chain and human exposure routes.

VDRs encompass active pharmaceutical ingredients (APIs), their metabolites, and impurities associated with the production process. These persistent contaminants infiltrate the tissues of food animals following therapeutic or prophylactic administration. Due to their multi-source nature and potential implications for human health, these residues pose a significant threat to food safety. As illustrated in Figure 2, a systematic classification of contamination pathways reveals four primary routes through which VDRs infiltrate the agri-food chain.

### 2.1. Feed Contamination

Feed represents a critical vector for VDRs in animal-derived foods. Risks primarily originate from two sources: illicit incorporation of veterinary drugs into feed formulations and historical reliance on antibiotic growth promoters (AGPs). Since the discovery of the growth-enhancing properties of antibiotics in 1946 [14], subtherapeutic doses of antibiotics have been systematically administered in intensive farming systems to increase growth rates, improve feed conversion efficiency, and prevent disease. AGPs have multiple benefits, including reduced morbidity/mortality from subclinical infections, enhanced weight gain, optimized production costs, improved reproductive performance, and superior meat quality [15]. By the 21st century, nearly 88% of U.S. grower-phase swine had received antibiotic-supplemented feed, reflecting widespread adoption in industrialized agriculture [15]. Furthermore, antimicrobials used during fermentation or processing of feed components (e.g., vitamins, distillers’ grains, and insect meal) may inadvertently contaminate finished feeds.

However, the global proliferation of antibiotic-resistant bacteria (ARB), particularly multidrug-resistant “superbugs,” has elevated AMR into a paramount public health crisis. For this reason, Sweden pioneered a ban on antimicrobial growth promoters in animal feed as early as 1986. Subsequently, the 1831/2003 regulation implemented by the European Commission in 2006 established the prohibition of using unnecessary antibiotics as growth promoters in livestock farming. However, due to the threat posed by AMR and its relationship with the use of antimicrobial drugs and heavy metals, as well as environmental concerns, the preventive use of antibiotics and ZnO were prohibited in January 2022 (Regulation (EU) 2019/6 on Veterinary Medicinal Products) and June 2022 (Regulation (EU) 2019/4 on Medicated Feed) [16,17,18,19]. Nevertheless, noncompliant feed producers continue illicit veterinary drug supplementation to meet market demands for accelerated growth and lean meat yields, often employing unregulated additives that result in hazardous drug accumulation in food animals.

Secondary contamination pathways can occur during the production, transportation, and storage of feed. Cross-contamination frequently occurs between batches processed using shared equipment, as antibiotic residues in feed can contaminate subsequent batches. This phenomenon, which is influenced by drug physicochemical properties, equipment design, and cleaning protocols, is particularly challenging to mitigate [20]. The ability of transport vehicles and storage facilities to retain antibiotic residues further exacerbates contamination risks. Eleni et al. [21] quantified this issue through Monte Carlo simulations and demonstrated that when antimicrobial-containing feeds constitute 2% of national production, approximately 5.5% (95% Confidence Interval: 3.4–11.4%) of the total annual feed output becomes cross-contaminated, with farm-level storage resulting in the highest contamination probability. Such antibiotic-contaminated feeds constitute a major reservoir of VDRs in animal products, posing significant threats to human health, food safety, and ecological security.

### 2.2. Irrational Use of Drugs

The irrational use of drugs is characterized by indiscriminate prophylactic administration, nonadherence to prescribed dosages and dosing schedules, an incorrect route or site of administration, excessive drug dosage at a single injection site, off-label use of drugs (unauthorized utilization in animals for which they are not approved, usage in other animal species for which they are suitable, treatment of conditions other than those specified), etc. [22], resulting in impaired excretion through normal metabolic processes in animals, leading to increased residual amounts or prolonged retention times. A survey in the Ghanaian poultry sector revealed tetracyclines as the predominant antimicrobial class, with 86% of farms implementing prophylactic drug regimens. Consequently, 24.3% of the sampled farms presented tetracycline residues in eggs, 92.9% of which continued commercial egg sales during the hen treatment phases. The study also documented regulatory breaches: 44.3% used disinfectants, whereas 17.4% and 2.6% administered two human-grade antibiotics off-label, violating usage protocols [23]. In low- and middle-income countries, the risk of antimicrobial misuse is further exacerbated by a lack of veterinary expertise, limited access to timely and accurate veterinary diagnostics, and weak regulation of pharmaceuticals. Farm operators often use antimicrobials on the basis of experience rather than professional consultation, primarily purchasing medicines from private veterinary pharmacies. From an economic policy perspective, small-scale farmers struggle to afford diagnostic costs, whereas governments may prioritize animal health and specific industries such as pig farming less because of budget constraints. For example, field observations in Uganda’s dairy industry revealed that although local veterinarians are aware of advanced diagnostic and treatment protocols, financial limitations prevent their implementation [24]. Notably, poultry farmers demonstrate marginally better antimicrobial stewardship than pastoralists do in terms of knowledge-attitude-practice metrics [25].

### 2.3. Too Short a Withdrawal Period

The withdrawal period is defined as the period from the discontinuation of drug administration in animals to approval for the slaughter or marketing of their products (such as milk and eggs). During this period, drug residues in the animal’s body are gradually metabolized and excreted, with residue levels decreasing below specified limits. The duration of the withdrawal period is influenced by various factors, including the animal species, type of drug, formulation, dosage, route of administration, distribution within the body, and farming environment [26].

Global regulatory fragmentation persists, as withdrawal periods are established on the basis of jurisdictional Maximum Residue Limits (MRLs). The outer packaging of veterinary drugs on the market generally indicates withdrawal periods. However, in actual application, factors such as disease heterogeneity, varying severity of illness, and differing drug dosages often result in actual withdrawal periods deviating from the standard guidelines. Therefore, to ensure meat products meet veterinary drug residue regulations, enterprises must conduct their own withdrawal period testing before slaughter. However, many small-scale farmers lack the knowledge and financial resources for such testing, prioritizing drug efficacy while neglecting the realities of drug resistance and withdrawal periods [27].

Noncompliance manifests as premature slaughter—whether intentional (due to economic incentives) or accidental (resulting from management errors)—leading to MRL violations. A Bangladesh study of 4200 postwithdrawal poultry samples detected 55% antibiotic residue exceedances, with fluoroquinolones (enrofloxacin: 38%; ciprofloxacin: 27%) demonstrating chronic noncompliance, revealing systemic governance failures in reconciling livestock productivity and food safety [27,28].

### 2.4. Environmental Pollution

Veterinary antibiotics enter the environment primarily through livestock excreta (urine/feces), wastewater effluent, improper drug disposal, pharmaceutical production discharge, and aquaculture runoff (Figure 2). Climate change and water scarcity drive the reuse of treated wastewater and livestock manure in agriculture, offering resource efficiency but introducing risks of antibiotic residues, resistance genes, and mobile genetic elements (MGEs) into ecosystems [29].

In livestock farming, 30–90% of administered antibiotics are excreted as active compounds or metabolites, with manure containing concentrations ranging from tens of mg/kg to higher levels [30]. Paul et al. [31] demonstrated cross-contamination via pasture, detecting phenylbutazone residues in grazing cattle weeks after initial exposure. Similarly, aquaculture discharges 70–80% of antibiotics into surrounding water and sediment due to low aquatic uptake [32]. These pollutants infiltrate agricultural systems via manure fertilization, wastewater irrigation, and biosolid reuse, contaminating soil and water through leaching or runoff [32]. Antibiotic emissions correlate directly with usage rates, with soil acting as the primary reservoir. In China, the annual emissions of 80 veterinary antibiotics (2010–2020) ranged from 23,110 to 40,850 tons, with 56%, 23%, and 18% of the total accumulated in soil, freshwater, and marine systems, respectively [33].

A wide range of antibiotics released into the environment through various channels are absorbed by plants, and certain antibiotics even accumulate within plants, where they enter the food chain through plant uptake. Hu et al. [34] employed a simplistic migration model to predict the accumulation of antibiotics in soil and discovered that vegetables absorb antibiotics in soil primarily through water transportation and passive absorption. The separate sampling and detection of antibiotics in leaves, stems, and roots revealed that the highest concentration of antibiotics was present in plant leaves, whereas the lowest concentration was in roots, indicating biological accumulation. Moreover, since the 1950s, antibiotics have been employed to control specific bacterial diseases in high-value fruits, vegetables, and ornamental plants. At present, the most commonly used antibiotics for plants are streptomycin and a small amount of oxytetracycline. For example, the US Environmental Protection Agency (EPA)-approved streptomycin and oxytetracycline applications combat citrus greening disease in Florida [35].

## 3. Human Exposure to Veterinary Drug Residues

The widespread use of veterinary antibiotics in livestock production, driven by growth-promoting agents and therapeutic interventions, has significantly heightened concerns over human exposure to residual antimicrobial compounds through animal-derived food products. Multimodal pathways exist for potential contact with these hazardous substances, including both direct and indirect environmental dissemination. Antibiotic residues are ubiquitously detected across diverse environmental matrices. In aquatic compartments, contamination affects groundwater aquifers, propagates through surface water systems, infiltrates municipal drinking water supplies, and persists in wastewater treatment effluents. Within terrestrial ecosystems, residues infiltrate river sediments, accumulate in agricultural soils, adsorb to atmospheric particulates, and even translocate into plant tissues (e.g., leafy vegetables). This environmental persistence creates a nested web of exposure risks. The primary routes include the ingestion of contaminated foodstuffs and drinking water, whereas the secondary pathways involve dermal absorption through contact with polluted soil/water or the inhalation of antibiotic-laden aerosols (Figure 2).

### 3.1. Food Intake

The global increase in antibiotic use in intensive livestock and aquaculture systems has rendered human exposure to VDRs through animal-derived foods virtually inescapable. Animal-derived foods serve as primary dietary sources of high-quality protein, lipids, and essential amino acids. These include meat (pork, beef, and lamb), poultry (chicken and duck), eggs, seafood (fish, crustaceans, and mollusks), and dairy products, constituting vital components of daily nutrition. Given this inevitability, mounting evidence indicates that animal-based foods represent the primary route for unintentional human intake of antibiotics.

The WHO established the Global Environment Monitoring System-Food Contamination Monitoring and Assessment Programme (GEMS/Food) in 1976. Participating institutions submit data on contaminant concentrations in food, and data centers are set up to assist national governments, the Codex Alimentarius Commission (CAC), and other bodies in assessing trends in food contamination [36]. Within this framework, the Joint FAO/WHO Expert Committee on Food Additives (JECFA) conducts risk assessments and develops recommendations for MRLs for veterinary drugs in various foods [37]. These recommendations are subsequently issued by the Codex Alimentarius Commission (CAC). This process provides a science-based international benchmark for countries to establish their own national standards [38,39].

Regulatory countermeasures, such as veterinary drug restrictions in the EU, US, and Japan, alongside China’s implementation of MRLs since the late 1990s, have yet to fully mitigate contamination. Key veterinary drugs (e.g., sulfonamides, tetracyclines) exhibit variations in their MRLs across major regulatory jurisdictions—such as the EU, the US, and China—as well as against the Codex Alimentarius standards (Table 1). These differences introduce complexity for global trade and uniform regulation. Persistent violations of veterinary drug regulations have been documented worldwide. For example, liquid chromatography-tandem mass spectrometry analysis of 146 poultry samples from Fujian, China, detected 39 antibiotics (quinolones, tetracyclines, and sulfonamides) in 47.3% of samples, with 11.6% exceeding MRLs [40]. Additionally, a study conducted at slaughterhouses in Ethiopia reported that 10% of kidney samples and 3.3% of muscle samples exceeded the MRLs for oxytetracycline [41]. In addition to animal products, environmental dispersion facilitates the uptake of antibiotics in plant-based foods. Analysis of commercial vegetables (lettuce, tomatoes, cauliflower, and broad beans) revealed seven antibiotics (16 targeted) at 0.09–3.61 ng/g fresh weight [42], confirming that the produce was a secondary exposure vector (Figure 2).

JECFA conducts global health evaluations to manage these risks. It establishes acceptable daily intake (ADI) thresholds, which are based on toxicological data from chronic exposure studies. However, recent studies highlight ongoing challenges. For instance, an analysis of the UK market found that over 35% of 34 sampled animal-derived foods contained residues of amoxicillin, ampicillin, and enrofloxacin that exceeded their respective ADIs [47].

Acute exposure risks are also evident, as shown by past incidents and new threats. For example, high concentrations of drugs at injection sites in livestock can create “hotspots.” Consuming meat from these areas in a single meal may cause acute poisoning. This is what happened in European clenbuterol poisoning outbreaks, which were traced back to eating veal liver and beef [48]. Immediate health outcomes range from allergic reactions and gastrointestinal distress to life-threatening toxicity, emphasizing the dual necessity of chronic risk management and acute exposure prevention frameworks.

### 3.2. Potable Water

Economic growth has accelerated the production and consumption of agricultural antibiotics, leading to their increased discharge and pervasive presence in various water bodies, including surface water, groundwater, and even tap water. Antibiotic residues originating from livestock farming, aquaculture, and pharmaceutical or hospital effluents often survive conventional wastewater treatment processes and are subsequently released into the environment [49]. For example, along the northern Persian Gulf coast in Iran, antibiotics such as azithromycin, erythromycin, norfloxacin, and tetracycline have been detected in coastal waters, with average concentrations ranging from 1.21 to 51.50 ng/L [50]. Contamination is particularly acute near antibiotic manufacturing hubs. Investigations near major production zones revealed that well water in six adjacent villages was contaminated with high concentrations of ciprofloxacin, enrofloxacin, moxifloxacin, and trimethoprim, while nearby lakes contained ciprofloxacin and enrofloxacin at levels exceeding 6500 ng/L [51].

Although purification processes exist for contaminated water, antibiotics remaining in water may be difficult to remove, particularly in areas reliant on groundwater. Moreover, this contamination exhibits regional variations. In Baghdad, Iraq, ciprofloxacin was the most commonly detected antibiotic in treated water from two municipal water treatment plants [52]. In a comparative study in China, 58 antibiotics were identified in filtered tap water samples, with roxithromycin being the most prevalent. Meanwhile, 36 antibiotics were detected in locally sold foreign-brand bottled water, including elevated levels of dicloxacillin [53]. The widespread release of antibiotics into aquatic environments is a major driver behind the proliferation of antibiotic resistance genes (ARGs). This threat is amplified when ARGs are associated with MGEs, which facilitate horizontal gene transfer (HGT) and accelerate the spread of resistance [29].

### 3.3. Occupational Exposure

ARB and their associated genes can be transmitted to humans through multiple pathways, including direct animal contact, consumption of contaminated food, and exposure to the environment via wastewater or feces [54]. Exposure to microbe-rich livestock-associated environments can reshape the human skin microbiome and antibiotic resistance profile. Such exposure exceeding 5 h is sufficient to alter the microbiome and ARG composition on workers’ skin through microbial enrichment and ARG acquisition. These alterations become persistent once established and may spread to the general population via farm workers as carriers of resistant genes [55]. Exposure of livestock employees to subtherapeutic antibiotic doses represents a specific scenario for the development of resistant bacteria. Studies confirm poultry workers carry *Escherichia coli* (*E. coli*) resistant to gentamicin at rates 32 times higher than the general community population. Farm workers also face significantly increased risk of carrying multidrug-resistant *E. coli* [56]. Veterinary antibiotics can enter the human body through skin contact and/or inhalation during professional operations performed by livestock workers. Analyses of dust from pig farms, collected over two decades, have consistently detected multiple antibiotics (e.g., tylosin, tetracyclines), indicating a chronic inhalation risk for workers [57]. Biomonitoring confirms the resultant systemic exposure; antibiotic levels measured in poultry workers’ urine have been found to exceed both microbial inhibition thresholds and recommended daily intake limits [58].

Personnel in pharmaceutical production face analogous hazards. Workers involved in antibiotic manufacturing processes, especially those handling active powders, are routinely exposed to antimicrobial agents. Longitudinal studies link this occupational exposure to the development of multidrug resistance (MDR). For example, workers in antibiotic production facilities have been shown to harbor significantly higher rates of MDR bacteria compared to control groups, with resistance prevalence increasing over time [59].

## 4. Toxic Effects of Drug Residues in Food

Pharmacologically active compounds may pose certain latent health risks to humans, including toxicological hazards (acute and chronic toxicity, allergic reactions, and carcinogenic, teratogenic, and mutagenic effects) and microbial hazards (disruption of the equilibrium of the intestinal flora and induction of resistance by intestinal bacteria). The hazards demonstrate a dose–response relationship, indicating that specific degrees of harm arise only when VDRs accumulate to a certain extent within the human body.

### 4.1. Acute and Chronic Toxic Effects

The long-term consumption of veterinary drug-contaminated foods can cause acute or chronic toxicity when accumulated residues reach critical thresholds. Acute symptoms (e.g., nausea, vomiting, neurotoxicity, dizziness, and convulsions) may arise from high-dose exposure to fluoroquinolones [60]. Sulfonamides impair kidney function and hematopoiesis [61], whereas chloramphenicol overexposure risks fatal aplastic anemia [62]. Tetracyclines inhibit bone/tooth development by binding to calcium [63], and erythromycin can induce liver injury [64], whereas aminoglycosides (such as gentamicin) carry the risk of potential ototoxicity, encompassing both vestibular and auditory impairment [65,66].

VDRs also disrupt endocrine and hormonal systems, interfering with hormone synthesis or activity. This imbalance increases the risk of reproductive disorders, developmental abnormalities, and chronic diseases such as obesity, diabetes, and cardiovascular conditions [67,68]. Endocrine-disrupting effects include estrogen-induced feminization in males, skeletal malformations, and carcinogenic impacts on female reproductive organs [69].

### 4.2. Development of Bacterial Resistance

Antibiotic use in animal agriculture exceeds that in human healthcare. This practice enables antibiotic resistance to spread from animals to humans through multiple pathways. According to data reported by 87 countries in 2020, several bacteria causing life-threatening blood infections exhibit high levels of resistance. Simultaneously, increased resistance to therapeutic drugs has emerged in several bacteria among common community-acquired pathogens [70]. The persistent escalation of AMR levels constitutes an ongoing global public health emergency with the potential to trigger the next pandemic outbreak. In 2019 alone, antibiotic-resistant infections directly caused 1.27 million deaths, whereas drug-resistant pathogens contributed to nearly 50 million fatalities [71]. Projections indicate that AMR-related mortality could reach 10 million annually by 2050, surpassing cancer deaths [71], with current estimates attributing 5 million annual deaths globally to AMR [72]. Pathogens such as *Mycobacterium tuberculosis*, *Neisseria gonorrheae*, and the ESKAPE group (*Enterococcus faecalis*, *Staphylococcus aureus*, *Klebsiella pneumoniae*, etc.) exhibit panresistance to conventional therapies, exacerbated by agricultural antibiotic use that selects for resistant foodborne pathogens, a trend that is predicted to increase future food safety risks [73].

Environmental contamination plays a crucial role in the dissemination of AMR. Unregulated antibiotic discharge, coupled with inefficient wastewater treatment systems, allows residual antibiotics and ARGs to persist in ecosystems. Junjie et al. [74] identified 11 ARGs, one integron, and one transposon in water and soil near a Chinese pharmaceutical facility, with the abundance of downstream ARGs significantly exceeding that of upstream nonpolluted areas (*p* < 0.05). The study further confirmed significant positive correlations (*p* < 0.05) between MGEs and ARG abundance. HGT mechanisms enable pathogens to acquire resistance traits, propagating multidrug-resistant strains through environmental and biological vectors. Quinolone and carbapenem resistance genes were detected in animal feces without drug treatment, further indicating that the gut microbiomes of humans and animals serve as hotspots for ARG exchange [75].

Human exposure risks extend beyond agricultural settings through three interconnected routes: aquatic systems, food chains, and companion animals [76]. Irrational discharges and inadequate sewage treatment facilities allow antibiotics and resistance genes to infiltrate drinking water supplies. Antibiotics and ARGs may also transfer to soil through crop irrigation and animal manure composting, subsequently entering the food chain [32]. Lactic acid bacteria (LAB) used in food fermentation also serve as reservoirs for AMR genes. The same tetracycline, erythromycin, and vancomycin resistance genes found in clinical bacterial species have been detected in *Lactococcus* and *Lactobacillus* species isolated from fermented meat and dairy products [77].

Concurrently, the global rise in pet ownership creates new pathways for AMR transmission. Increased “contact” between companion animals and humans elevates infection risks and facilitates cross-transmission of AMR traits. Microorganisms transmitted from vertebrates—including livestock—to humans are estimated to constitute 60% of human pathogens [78]. Microorganisms can be transmitted through inhalation, ingestion, conjunctival contact, or physical contact. Veterinary antibiotic use often relies on empirical prescribing rather than antimicrobial susceptibility testing, potentially contributing to resistance in pathogens such as methicillin-resistant *Staphylococcus aureus* and carbapenem-resistant *Enterobacteriaceae* [79].

### 4.3. Causes of Dysbiosis of the Flora

Bacteria are the most representative members of the human gut microbiota, comprising over 1000 species, the majority of which are anaerobic. In the adult gut, approximately 10^14^ bacterial cells exist—ten times the number of human cells. Their combined genomes (termed the microbiome) contain over 5 million genes, exceeding the host’s genetic potential by two orders of magnitude [80]. Extensive data describe the gut microbiome’s role in host physiological processes, including metabolism, skeletal development, nutritional and immune processes, and suppression of growth by potential pathogenic microorganisms within the gut. Indeed, the metabolic capacity of the gut microbiota equals that of the liver, allowing it to be considered an additional organ. The gut microbiota also modulates inflammatory responses in the lungs. Studies reveal that antibiotic-disrupted microbiota alter the lung’s inflammatory response to lipopolysaccharide (LPS) stimulation [81]. The gut microbiota can also directly and indirectly modulate the host serotonin (5-hydroxytryptamine; 5-HT) system. 5-HT acts within the gastrointestinal tract to regulate numerous intestinal functions, including motility and secretion. Additionally, the gut microbiota produces numerous microbial metabolites that can indirectly influence the host’s endocrine system in multiple ways [82].

While antibiotics combat pathogens, broad-spectrum antibiotics indiscriminately eliminate commensal microbiota, leading to microbial imbalance (dysbiosis) and triggering systemic consequences [83]. For example, low-dose erythromycin/azithromycin therapy impairs microbial carbohydrate metabolism and short-chain fatty acid synthesis, correlating with alterations in systemic immune biomarkers (IL-5, IL-10, and MCP-1) and metabolic regulators (5-HT and C-peptide) [82]. Studies demonstrating a concentration-dependent reduction in bacterial richness proportional to moxifloxacin dosage confirm that antibiotic-induced diversity loss is concentration-dependent [84].

Antibiotic use also impacts gut microbial community composition. Studies reveal that short-term combined meropenem/gentamicin/vancomycin therapy promotes proliferation of *Enterobacteriaceae* and *Prevotella* species while reducing *Bifidobacteria* and decreasing butyrate production—metabolic shifts potentially carrying carcinogenic implications given the microbiome’s role in colorectal cancer development [85]. Early-life exposures exert lasting impacts: neonates exposed to intrapartum maternal antibiotics display reduced *Bacteroidetes* abundance and enriched *Firmicutes* (particularly Clostridia and Enterococci), establishing dysbiotic trajectories [86]. These interindividual variabilities in antibiotic susceptibility underscore the complexity of microbiota–host interactions, positioning gut microbial integrity as both a therapeutic target and a vulnerability in modern pharmacotherapy [87].

### 4.4. Carcinogenic, Teratogenic, and Mutagenic Effects

In recent years, scientists have demonstrated that exposure to low doses of antibiotics is correlated with numerous human health issues, including obesity, carcinogenicity, reproductive effects, teratogenicity, and mutagenicity. The genetic toxicity and carcinogenicity of certain antibiotics pose safety hazards to target animals and humans. For example, Cao et al. [88] reported that the long-term and persistent use of antibiotics can lead to the proliferation of colonic polyps, presenting a potential threat to the development of cancer. The excessive intake of estrogen residues by women may lead to breast cancer, ovarian cancer, and endometrial cancer; aflatoxin and tetracycline can induce liver cancer [89]. Mutagenic drugs contain certain chemicals, such as alkylating agents and DNA base analogs, which can induce mutagenic activity and potentially cause gene mutations or chromosomal breaks, thereby influencing human fertility [89]. Streptomycin, kanamycin, and tetracycline are regarded as teratogenic and must be thoroughly avoided during pregnancy since they interfere with fetal development. These drugs can result in hearing impairment and developmental inadequacies [90].

### 4.5. Anaphylaxis

When humans ingest animal food containing allergenic VDRs, the antigenic molecules in veterinary drugs might bind to immunoglobulin E in the human body. This binding process triggers the release of inflammatory mediators, including histamine, by immune cells, which leads to the occurrence of allergic symptoms in the skin and respiratory system, as well as delayed hypersensitivity reactions. Some antibiotics, such as penicillin and sulfonamides, can induce allergic reactions in certain individuals, even anaphylactic shock. Penicillin-type drugs are prone to eliciting allergic reactions, ranging from mild skin reactions and contact dermatitis to anaphylactic shock, which can result in death [91]. Sulfonamide drugs may impair the hematopoietic function of humans. Tetracyclines cause specific allergic reactions, such as allergic rashes, dermatitis, and respiratory difficulties [92].

## 5. Mitigation Strategies for Drug Residues in Animal-Derived Foods

Controlling VDRs in animal-derived foods remains a persistent and complex challenge within the current food safety framework. Addressing this issue requires concerted efforts across multiple fronts. Animal producers must adopt scientifically sound husbandry practices and comply with relevant regulations; governments must strengthen grassroots oversight and provide multifaceted policy support; and researchers must dedicate themselves to developing superior alternatives to antibiotics.

### 5.1. Rational Use of Antibiotics in Animal Farming

Vaccination is a cost-effective method for reducing the incidence of major diseases and decreasing antibiotic use. It prevents the spread of animal epidemics, thereby reducing opportunities for antibiotic administration [72]. The judicious selection of veterinary drugs is equally critical. Substandard or counterfeit antibiotics—which often have problems such as inaccurate active ingredient content or mislabeled information—can compromise therapeutic efficacy and exacerbate resistance risks [93]. During disease outbreaks, etiological treatment should be implemented through pathogen-specific diagnostics (e.g., culture and susceptibility testing) to match antibiotics with causative microorganisms, minimizing unnecessary exposure. Strict adherence to prescribed treatment durations and dosages (individualized based on animal age, weight, and health status) is essential. Additionally, synergistic drug combinations can broaden antimicrobial spectra, reduce dosages, and remain effective against certain resistant strains. For example, low-dose felodipine enhances gentamicin’s activity by inhibiting expression of aminoglycoside resistance-associated proteins (aacA-aphD) [94,95]. Combination therapy may also be considered based on pharmacokinetic properties. For instance, when quinapimide is combined with carbomer, the latter acts as a drug retention polymer, forming a stable complex with quinapimide in the gastrointestinal tract. This significantly delays drug excretion, prolonging its residence time at the site of action [96]. Alternatively, pharmacodynamic effects may be considered, such as vitamin D mitigating the nephrotoxicity induced by levofloxacin [97].

### 5.2. Strengthening the Supervision and Education of Veterinary Drugs

Farmers’ understanding of veterinary drugs, their awareness of the importance of safe veterinary drugs, and their awareness of government regulation all influence their standardized business practices. Surveys indicate that farmers’ understanding of veterinary drugs is the most critical factor affecting their consumption behavior [98]. Therefore, governments, industry associations, and environmental organizations should actively utilize modern platforms (such as short videos) and traditional outreach methods (such as public notices and radio broadcasts) to enhance farmers’ understanding of veterinary drugs, including their proper use, withdrawal periods, and knowledge of livestock and poultry diseases. Develop preferential policies to encourage farmers to participate in industrialized farming, incentivize industrial organizations to actively conduct various technical training programs, and foster a favorable producer market environment. Establish communication and cooperation platforms connecting farmers, slaughter/processing enterprises, and markets to regulate livestock producers’ behavior through market organization [98,99].

Create economic incentives for responsible drug use. Increase subsidies for pollution-free and green veterinary drugs while strengthening penalties for unsafe veterinary drug practices. Often, livestock producers’ non-compliant drug use is driven by short-term gains. By aligning livestock producers’ long-term interests with responsible drug practices through direct economic benefits, regulatory authorities can more effectively motivate producers.

Establish a full-chain traceability system for antibiotic production, sales, and farm operations. Veterinary drug distributors must improve inventory management records and disposal procedures to reduce expiration rates, ensuring drug quality and traceability of sources and destinations [100]. Establish online prescription platforms, electronic antibiotic records, and withdrawal period monitoring tools to ensure transparency in antibiotic distribution and usage. Livestock producers must standardize farming practices and maintain comprehensive records throughout the process, including the use of ear tags for livestock identification, breeding records, and animal quarantine documentation. Ensure all animal medication histories are traceable [101]. Implement a registration system and credit rating system for veterinary drug distributors and livestock farmers, fostering mutual oversight to create a closed-loop system.

### 5.3. Developing Alternative Varieties of Antibiotics

The Third Global High-Level Ministerial Meeting on AMR agreed to reduce the total use of antimicrobials in animals and agriculture by at least 30–50% by 2030, incentivizing national and global efforts [102]. Preserve critically important antimicrobial agents for human medicine and cease the use of medically important antimicrobials for non-veterinary medical purposes, including for promoting animal growth. Current efforts to replace traditional antibiotics primarily focus on identifying environmentally friendly alternatives. Key areas of ongoing research include probiotics, bacteriophages, organic acids, antimicrobial peptides, botanical extracts, nanoparticles, and Abs (Table 2).

#### 5.3.1. Probiotics

Probiotics can regulate the abundance of gut microbiota and the colonization of pathogens. Live probiotic microorganisms help increase gut microbial diversity, promote the growth of beneficial bacteria, exclude pathogens, and modulate immune function and detoxification. Additionally, probiotics improve gut-related functions such as promoting digestive enzyme secretion, nutrient absorption, and short-chain fatty acid production, thereby enhancing growth performance and feed conversion efficiency [103]. The efficacy of probiotics is often constrained by strain, dosage, and host dependency [117]. Kang et al.’s study [104] demonstrated through in vivo and in vitro experiments that specific probiotic strains combined with grapefruit seed extract can alleviate *Clostridium difficile* infection by modulating the gut microbiota.

#### 5.3.2. Phage

Once a bacteriophage attaches to its bacterial target, it injects its genetic material into the bacterium and utilizes the bacterium’s replication machinery to generate new phage particles. This process, known as the lytic cycle, ultimately concludes with the lysis or rupture of the bacterial cell, releasing new phages capable of infecting additional bacteria [118]. The phage’s ability to self-replicate at the site of infection represents a distinct advantage, as it may reduce the need for repeated dosing. The high specificity of phages minimizes disruption to the host microbiome. However, this also necessitates a thorough understanding of the bacterial pathogen to select the appropriate phage for treatment, making the therapeutic process more complex compared to the broad-spectrum efficacy of traditional antibiotics. Additionally, phages may be inactivated by gastric acid or the immune system, making systemic administration challenging [105]. Molendijk et al. [106] compared phages with the antibiotic fusidic acid in treating burn wound infections, finding phages demonstrated superior potential to antibiotics for such localized wounds.

#### 5.3.3. Organic Acids

Given its advantages of being nonpolluting, nonresistant to antibiotics, and leaving no residues, the EU allows the use of acidifiers or organic acids and their salts in poultry farming. The incorporation of organic acids into poultry diets aids in the production of prebiotics and probiotic lactic acid bacteria. Organic acids can serve as alternatives to AGPs, positively impacting the production efficiency and intestinal health of livestock and poultry. By supplementing organic acids, one can improve growth performance indicators and carcass characteristics, lower the intestinal pH, increase the production of gastric proteases, increase the digestion and absorption of nutrients, stimulate immune responses, and suppress the proliferation of pathogenic bacteria [107,119]. However, the efficacy of organic acids can be influenced by dietary composition, particularly the buffering capacity of the feed, which may necessitate higher doses that could, in turn, reduce feed palatability. Wang et al. [108] demonstrated that the combination of organic acids and medium-chain fatty acids has significant antibacterial effects and alleviates early weaning diarrhea in piglets by regulating the intestinal microbiota and strengthening intestinal barrier function, revealing a practical strategy to increase their effectiveness.

#### 5.3.4. Antimicrobial Peptide

Antimicrobial peptides are host defense peptides derived from the innate immune system of various organisms, including animals, plants, bacteria, and fungi. They exhibit their antimicrobial activities through diverse mechanisms, such as disrupting bacterial cell walls and membranes, functioning intracellularly, and inhibiting biofilm formation [120]. This multi-mechanism action reduces the potential for inducing bacterial resistance. Additionally, studies indicate that antimicrobial peptides possess immunomodulatory functions, offering multifaceted therapeutic potential [120]. Xia et al. [109] conducted a zebrafish culture experiment where fish were fed diets enriched with antimicrobial peptides or antibiotics to assess their effects on gut microbiota and antibiotic resistance profiles. Results showed lower absolute abundance of ARGs in the antimicrobial peptide-treated group compared to the antibiotic-treated group. This finding highlights antimicrobial peptides’ potential in inhibiting resistance development. However, their poor stability and high production costs somewhat limit their application [110].

#### 5.3.5. Botanical Extracts

Chinese herbal medicines are widely sourced, affordable, and have minimal side effects. They are rich in nutrients such as saponins, proteins, amino acids, minerals, and vitamins, as well as bioactive compounds including polysaccharides, flavonoids, alkaloids, organic acids, and essential oils [111]. Compared to traditional antibiotics, natural herbal extracts exhibit broad-spectrum antibacterial activity and low resistance rates. However, the content of active components in herbal extracts may vary significantly between batches. Furthermore, optimal dosage regimens and precise mechanisms of action often require further investigation. Studies have demonstrated that dietary supplementation with Achyranthes japonica extract can enhance various production parameters in broiler chickens in a dose-dependent manner, including promoting growth performance, improving nutrient digestibility, and regulating cecal microbiota [111]. Similarly, Bendowski et al. [112] reported that Silybum marianum supplementation in broilers reduced footpad lesion incidence, enhanced welfare, improved meat quality, and increased antioxidant activity.

#### 5.3.6. Nanoparticles

Nanotechnology, as an emerging science, is being widely applied across multiple research fields, including medicine, delivering significant benefits. For instance, metallic nanoparticles such as silver (Ag), gold (Au), silver oxide (Ag_2_O), zinc oxide (ZnO), titanium dioxide (TiO_2_), calcium oxide (CaO), copper oxide (CuO), and magnesium oxide (MgO) have demonstrated immense potential in the field of antimicrobial applications [110]. Regarding the antibacterial mechanisms of these metal nanoparticles, current research has revealed multiple potential pathways. These mechanisms include, but are not limited to: direct damage to nucleic acids; disruption of cell walls and membranes through the formation of pits and pores; interference with normal cellular structure and function; induction of protein oxidation, interruption of electron transport processes, and inhibition of cell division; promotion of reactive oxygen species (ROS) formation, leading to enzyme degradation or inhibition; inactivation or leakage of cellular substances; and disruption of flagella, cell integrity, and the cytoplasmic matrix [121]. This multi-mechanistic mode of action significantly reduces the development of microbial antibiotic resistance. However, research on nanotechnology remains limited, and key biosafety concerns regarding its potential toxicity, environmental persistence, and bioaccumulation remain incompletely understood [113]. Raza et al. [114] evaluated the potential of zinc oxide and copper oxide nanoparticles as alternatives to florfenicol in treating fowl cholera in chickens. This study provides important reference data for assessing the practical applicability and safety of nanoparticles.

#### 5.3.7. Antibodies

Abs are primarily produced in mammals, exhibiting high specificity and safety. They can directly target bacterial surfaces or indirectly neutralize bacterial toxins and virulence factors causing infection, offering a useful alternative for treating bacterial infections. Research by El-Kafrawy et al. demonstrated that egg yolk IgY Abs generated from immunized chickens effectively treat bacterial infections in animals and humans [115]. However, Abs are characterized by high production costs, limited targeting scope, and ineffective oral administration. Emelianova et al. demonstrated that combining highly diluted specific Abs formulations with antibiotics significantly improved survival rates in mice infected with *Klebsiella pneumoniae*, with efficacy comparable to the reference drug gentamicin [116]. This combination strategy leverages the specificity advantage of Abs while partially reducing costs.

## 6. Food Safety Assurance Measures

**Figure 3:**
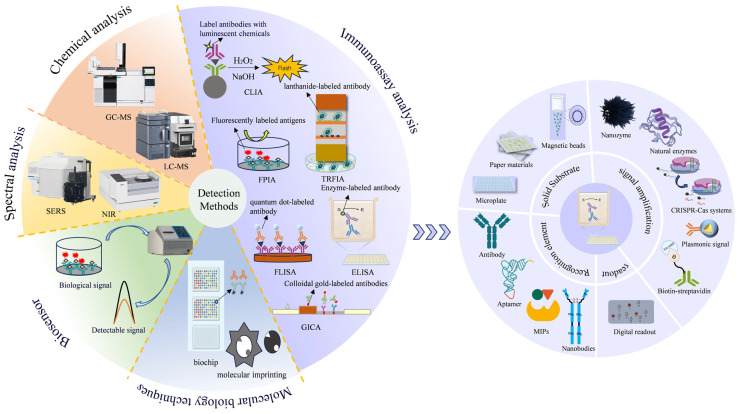
A summary of veterinary drug residue detection methods in the food chain. Detection methods are color-coded: orange, chemical analysis; purple, immunological analysis; blue, molecular biology techniques; cyan, biosensors; yellow, spectroscopic analysis. The arrow from the purple section (immunological analysis) points to an enlarged view of the improvements in ELISA derivative techniques; these improvements encompass four main aspects: solid substrates, recognition elements, signal amplification strategies, and readout methods.

The use of veterinary drugs in intensive farming inevitably leads to persistent drug residues throughout the food chain, posing significant threats to public health through bioaccumulation and chronic exposure pathways. Precise identification and quantitative detection of residual compounds—including antibiotics, antiparasitics, and growth promoters—in meat, eggs, dairy products, and related items necessitate continuous advancement in analytical methods. In recent years, the field of veterinary drug analysis has witnessed continuous innovation, leading to the development of more efficient, highly sensitive, accurate, and user-friendly detection platforms (Table 3 and Figure 3), which have steadily enhanced residue detection capabilities.

### 6.1. Chemical Analysis Method

Gas chromatography (GC) and liquid chromatography (LC) are key techniques for separating VDRs in complex samples. Notably, in modern confirmatory analysis, these two techniques are typically coupled with mass spectrometry (MS) detectors. Chromatography–mass spectrometry techniques (such as gas chromatography–mass spectrometry (GC–MS) and liquid chromatography–mass spectrometry (LC–MS)) can simultaneously provide the retention time and characteristic mass spectrum of target compounds, thereby achieving qualitative confirmation and precise quantification of residues.

According to the EU Commission Implementing Regulation (EU) 2021/808 [147] and Codex Alimentarius guidelines (CAC/GL 71-2009) [148], methods capable of providing sufficient structural information, such as GC–MS/MS and LC–MS/MS, are stipulated as analytical methods suitable for substance confirmation. This makes them an indispensable core confirmatory means in global official veterinary–sanitary control and trade arbitration. Other chromatographic techniques or immunological methods are primarily used for high-throughput screening or scientific research, and their positive results require further confirmation via mass spectrometric methods.

#### 6.1.1. Gas Chromatography

GC is primarily employed for the detection and analysis of volatile or semivolatile organic compounds. In GC, the sample is introduced into the heated vaporization chamber via an injector, and the components of the sample are vaporized and conveyed by the carrier gas. The separation of diverse components is accomplished through the separation effect of the chromatography column, and the detection signal is obtained at the detector. GC boasts the advantages of high separation efficiency, high sensitivity, and rapid analysis speed and is frequently utilized to detect residues of pesticides and volatile organic pollutants. The detectors linked to GCs mainly include nitrogen-phosphorus detectors, electron capture detectors, and mass spectrometry detectors. Shuyu et al. [122] developed an innovative column-pretreatment derivatization gas chromatography–tandem mass spectrometry (GC–MS/MS) technique for the precise determination of decoquinate residues in chicken tissue. Before the derivatization step, the polar functional groups within the compound structure were eliminated by hydrogenation reduction, which not only decreased the molecular weight but also effectively increased the volatility of the decoquinate, creating favorable conditions for subsequent chromatographic analysis.

#### 6.1.2. Liquid Chromatography

LC and its advanced derivatives, including high-performance liquid chromatography (HPLC) and ultrahigh-performance liquid chromatography (UHPLC), are widely employed for detecting nonvolatile or thermally labile compounds. These techniques separate sample components under high-pressure column conditions through differential partitioning between mobile and stationary phases, offering high throughput, sensitivity, and specificity for VDR analysis. The integration of UHPLC with tandem mass spectrometry (UPLC-MS/MS) further enhances analytical capabilities by combining chromatographic resolution with mass spectrometry selectivity, enabling rapid identification of residues, metabolites, and derivatives in complex matrices. High-resolution mass spectrometry (HRMS) platforms, such as quadrupole-linear ion trap (Q-LIT) and Orbitrap-based systems, provide unparalleled mass accuracy (<1 ppm) and resolution (>100,000 FWHM), facilitating nontargeted screening and structural elucidation of veterinary drugs. This capability was demonstrated by Vardali et al. [123], who utilized ultrahigh-performance liquid chromatography coupled with quadrupole time–of-flight tandem mass spectrometry (UHPLC–QTOF–MS) to analyze 20 VDRs (tetracyclines, quinolones, and sulfonamides) in European sea bass tissues. Similarly, Lu et al. [124] established a PRiME extraction workflow coupled with UHPLC-Q-LIT/electrostatic field orbitrap HRMS for multiclass detection (sulfonamides, β-lactams, and macrolides) in sheep milk.

### 6.2. Immunological Technique

Immunological analysis is a form of biochemical analysis that exploits the specific binding reaction between antigens and Abs. It typically exhibits high selectivity and low detection limits and is extensively employed for the determination of various antigens, semiantigens, or Abs. It is generally classified into several methods, such as enzyme-linked immunosorbent assay (ELISA), colloidal gold immunoassay (CGIA), chemiluminescence immunoassay (CLIA), fluorescence polarization immunoassay (FPIA), quantum dot fluorescence immunoassay (FLISA), and TRFIA.

#### 6.2.1. Enzyme-Linked Immunosorbent Assay

ELISA is a widely used diagnostic technique that detects and quantifies target molecules through the specific binding of Abs and antigens. Traditional ELISA employs microplates as solid substrates and Abs as recognition elements, but its limitations—such as operational complexity, high costs, and limited sensitivity—have driven innovations in its four core components: solid substrates, recognition elements, signal amplification systems, and readout methods (Figure 3) [149].

To improve portability and analytical efficiency, paper-based analytical devices and magnetic bead (MB) systems have emerged as viable alternatives to conventional microplate platforms. A notable example is the nanofiber membrane-integrated paper-based ELISA system, which facilitates rapid visual identification of trace chloramphenicol through enhanced capillary action [125]. Concurrently, MB-based platforms exhibit superior reaction kinetics owing to their high surface-to-volume ratio, enabling efficient biomolecular interactions [149].

Recognition elements have expanded beyond traditional Abs. Aptamers, owing to their low cost and stability, achieve quantitative chloramphenicol detection in fish at 16 pg/mL [126]. Nanobodies derived from camelid Abs combine high stability and sensitivity, such as horseradish peroxidase (HRP)-labeled nanobodies, which detect enrofloxacin in milk at a concentration of 6.48 ng/mL [127]. Molecularly imprinted polymers (MIPs), synthetic Ab mimics, offer robustness and cost-effectiveness, even outperforming natural Abs in some applications [150].

Signal amplification systems have evolved to address the limitations of natural enzymes such as HRP. Nanocatalysts (e.g., Au-Pt/SiO_2_ nanoparticles), CRISPR–Cas systems, biotin–streptavidin complexes, and plasmonic resonance strategies increase sensitivity and reduce reliance on unstable enzymes under extreme conditions. Readout methods have shifted from analog approaches to digital approaches. Digital ELISA partitions samples into tens of thousands of microreactors for single-molecule counting, achieving 1000-fold higher sensitivity than traditional methods do [151]. However, its application to small molecules (e.g., haptens) remains unreported [149,151].

Advancements in various aspects of traditional ELISA technology have expanded its applications in resource-limited settings and veterinary drug surveillance. However, challenges persist in applying digital ELISA to small molecule detection, underscoring the need for continued innovation [149].

#### 6.2.2. Colloidal Gold Immunoassay Assay

When colloidal gold (where gold chloride is reduced and polymerized to form gold particles of specific sizes) is used as a tracer, it combines with macromolecular substances such as proteins and is detected through immune reactions. This technology, in addition to its facile operation, rapid response, and short time consumption, also possesses the merits of low cost and high specificity and is particularly suitable for field detection. Haiping et al. [128] employed CGIA to achieve rapid, onsite, and semiquantitative detection of tetracycline in seawater. They achieved a visible limit of detection (vLOD) of tetracycline in seawater at 20 µg/L, with results observable by the naked eye within 10 min. This method remains unaffected by variations in sea temperature, pH, and salinity. Additionally, Hong et al. [129] created a colloidal gold immunoassay strip for detecting aldicarb in agricultural products and the environment. Their strip exhibited excellent performance, accurately identifying positive samples. The detection limit was set at 30 μg/kg, and the average recovery rate of pirimiphos-methyl in spiked samples ranged between 80.4% and 110.5%.

#### 6.2.3. Chemiluminescence Immunoassay Assay

CLIA technology integrates high-sensitivity chemical luminescence detection with highly specific immune reactions. The primary principle of this technology lies in marking the chemical luminescence substance on the antigen (or Ab) or acting on the chemical luminescence substrate with enzymes. Under catalytic and oxidative action, the chemical luminescent substance is excited from the ground state to the excited state, which is unstable. The luminescent substance then reverts to the ground state and releases photons. Since the intensity of the light signal is linearly correlated with the concentration of the analyte within a certain range, an optical detection system can be employed to quantitatively detect the light signal and determine the content of the analyte. Wu et al. [130] designed a chemiluminescence enzyme immunoassay (CLEIA) for the quantitative measurement of sulfamethoxazole. This method has a detection limit of 3.2 pg/mL, a linear range of 10–2000 pg/mL, intraday and interday precisions below 13% and 18%, respectively, and a recovery rate ranging from 85% to 105%. Furthermore, Yu et al. [131] introduced a CLEIA based on the HRP-luminol-hydrogen peroxide (H_2_O_2_) chemical luminescence system for the high-sensitivity detection of enrofloxacin. Compared with ELISA and HPLC, CLEIA has significant advantages, enabling high-throughput and real-time detection.

#### 6.2.4. Fluorescence Polarization Immunoassay Assay

FPIA is a relatively mature quantitative immunoassay technique. In a solution, a known concentration of fluorescently labeled antigen and the target (antigen) compete for binding with a certain amount of Ab. The fluorescently labeled antigen molecules are small and rotate rapidly, resulting in a low fluorescence polarization intensity. However, when the labeled antigen binds to the Ab to form an antigen–Ab complex, it rotates slowly, causing a high fluorescence polarization intensity. In the competitive process, the more target molecules there are, the more they bind to the Ab, and the less the Ab binds to the labeled antigen, leading to a lower fluorescence polarization intensity. By utilizing the relationship between the labeled antigen and the fluorescence polarization intensity, the number of target molecules in the solution can be calculated. Liuchuan et al. [132] created nine tracers by linking various haptens with fluorescein. These tracers were then matched with four monoclonal antibodies (mAbs), and the optimal tracer–mAb pair was selected to establish a high-sensitivity FPIA for detecting amantadine in chicken meat. During actual testing of chicken meat samples, the detection limit was found to be 0.9 μg/kg, with a recovery rate ranging from 76.5% to 89.3%. Dong et al. [133] synthesized ten fluorescently labeled ractopamine derivatives, known as tracers, and developed a rapid FPIA for detecting ractopamine in pork via a polyclonal Ab specifically prepared for ractopamine. The detection limit of this method was 0.56 μg/kg, and the recovery rate ranged from 74.8% to 86.6%.

#### 6.2.5. Fluorescence Immunoassay Assay

Quantum dots, abbreviated as QDs, are semiconductor quantum dot nanocrystals with dimensions ranging from 1 to 10 nm. They possess advantages such as color tunability, low photobleaching, intense fluorescence, a narrow emission spectrum, and a wide excitation spectrum, thus rendering them promising fluorescent probes. Through the combination of quantum dots with immunoanalytical techniques, the signal can be amplified, leading to high sensitivity, and these methods have been widely applied in the fields of environmental and food safety. For example, Xin et al. [134] presented a fluorescence immunoassay utilizing quantum dot fluorescence for the precise quantification of florfenicol residues in animal-derived food and feed. This method exhibited exceptional sensitivity, with a detection limit of 0.3 ng/mL and a quantitative range spanning from 0.6 to 30.4 ng/mL. In a separate study, Erqun et al. [135] integrated a multicolor QD-based immunofluorescence technique with an array analysis method to facilitate simultaneous, sensitive, and visual detection of streptomycin, tetracycline, and penicillin G in milk.

#### 6.2.6. Time-Resolved Fluoro Immunoassay Assay

TRFIA technology is a marker immunoassay technique that emerged in the 1980s. It utilizes lanthanide elements to label antigens or Abs and measures fluorescence by employing time-resolved technology on the basis of the luminescence characteristics of lanthanide chelate complexes. Through the detection of both wavelength and time parameters, effective signal discrimination can be accomplished to eliminate nonspecific fluorescence interference, thereby significantly enhancing the analytical sensitivity. The amount of attention it has received in the field of VDR detection has increased annually. A strip test based on time-resolved fluorescent microsphere immune probes (TRFMs-LFIAs) has been developed for the simultaneous detection of ceftiofur and its metabolite desfuroylceftiofur, with LODs of 0.97 ng/mL and 0.41 ng/mL, respectively, and IC_50_ values of 12.92 ng/mL and 12.58 ng/mL, respectively [136].

### 6.3. Spectrum Technology

Spectral technology uses the characteristic features of emission, absorption, or scattering spectral series of various chemical substances to detect and identify them. It is highly sensitive, easy to use, and requires no laboratory environment to achieve high-efficiency and high-precision online detection. The applications of spectral technology in the detection of VDRs primarily encompass surface-enhanced Raman spectroscopy (SERS) and near-infrared spectroscopy (NIRS).

#### 6.3.1. Surface-Enhanced Raman Spectroscopy

Raman spectroscopy is a nondestructive analytical method that enables the examination of a sample’s chemical composition, phase and morphological characteristics, crystallinity, and intermolecular interactions. It can rapidly and quantitatively analyze the components of diverse substances. In surface-enhanced Raman scattering, when target molecules adsorb onto solid substrates featuring metal nanostructures on the surface or onto metal nanoparticles such as gold and silver, the plasmon resonance effect of the metal surface can significantly enhance the Raman scattering signal of the molecules, with a strength that can be enhanced by 10^6^ to 10^15^ times in comparison with normal Raman spectra. It is capable of quantitatively identifying ultralow-concentration molecules in complex biological mixtures and providing comprehensive information. The sensitivity can reach the level of single molecules, facilitating rapid and nondestructive analysis of trace and ultratrace samples. Gao et al. [137] developed a solid-phase extraction (SERS) approach for detecting multiple antibiotic residues in dairy products. This method offers high sensitivity and throughput for identifying sodium tetracycline, sulfapyridine, and benzathine penicillin, with detection limits as low as 2.237, 2.644, and 4.662 ppb, respectively. In another study, Shuai et al. [138] utilized SERS in conjunction with spectral pretreatment techniques to detect nitrofurantoin in honey. By refining the experimental process and enhancing the spectral calibration method, they achieved a minimum detection limit of 0.1321 mg/kg for nitrofurantoin.

#### 6.3.2. Near-Infrared Spectroscopy

NIRS is a significant analytical technique with a wavelength range between visible light and mid-infrared spectra, typically delineated as 780 nm to 2500 nm. This spectral domain primarily registers the vibrational information of hydrogen-containing groups (such as C-H, O-H, and N-H), thereby rendering NIRS highly sensitive and precise for the analysis of organic compounds and biomolecules. Presently, NIRS has been applied and promoted in diverse analytical fields because of its rapid analysis speed, high detection efficiency, excellent test reproducibility, and nondestructive sampling. It can not only circumvent the problems of complex operation and environmental pollution in the sample pretreatment process of conventional detection but also satisfy different detection requirements, such as onsite detection and multiparameter simultaneous detection. Conceicao et al. [139] applied Fourier transform NIRS to identify antibiotic residues in milk and successfully differentiated various antibiotics dissolved therein. Moreover, Wu et al. [140] utilized NIRS alongside partial least squares regression to detect tetracycline residues in milk, achieving a sensitivity of 5 micrograms per liter, with a data model correlation coefficient exceeding 0.89.

### 6.4. Biosensor Technology

Biosensors typically consist of a recognition element (or receptor) and a transducer element. A biosensor generally comprises a recognition element (or receptor) and a transducer element. It is a specialized device that employs a biological sensing unit, relying on component recognition, to convert recognizable biological signals into detectable electrical, optical, acoustic, or thermal signals through appropriate energy conversion principles. Additionally, it adopts suitable techniques to ensure high selectivity during signal amplification for the target object. Owing to their strong specificity, rapid analysis speed, high precision, low cost, portability, and flexibility, biosensors are extensively utilized. Depending on the type of transducer element used, biosensors can be classified into electrochemical, semiconductor, optical, thermistor, and piezoelectric biosensors, which are applicable for analyzing indicators such as toxic and harmful substances, food components, food additives, and food freshness [152]. Peng et al. [141] developed a multiplexed lateral flow immunoassay, termed the AuNCs-MLFIA sensor, which uses highly luminescent green gold nanoclusters. This sensor can detect samples without prior treatment in just 18 min. Furthermore, the AuNC-MLFIA sensor can be integrated with a portable fluorescence reader for quantitative detection. The IC_50_ values for clenbuterol and ractopamine were 0.06 and 0.32 μg/L, respectively, with detection limits of 0.003 and 0.023 μg/L. Zhang and colleagues [142] crafted a highly sensitive amperometric immunosensor by integrating zinc sulfide quantum dots (ZnSQDs) and polyaniline nanocomposites with gold electrodes and clenbuterol Abs. This biosensor exhibited a remarkable detection limit of 5.5 pg/mL at an operating potential of 0.21 V versus Ag/AgCl.

### 6.5. Molecular Biological Technique

As traditional analytical techniques such as chromatography and mass spectrometry continue to evolve, molecular biology techniques are being increasingly employed in food testing. They play an increasingly significant role in the detection of pesticides and VDRs, the identification of genetically modified products, the detection of allergens, and the discrimination of pathogenic microorganisms. Among the molecular biology techniques utilized in the domain of VDR detection are biological chip technology and molecular imprinting technology.

#### 6.5.1. Biochip Technology

Biosensor technology leverages the specific interactions between biomolecules to construct a miniaturized biochemical analysis system. By employing microfabrication and microelectronic techniques, high-density immobilization of DNA, antigens, Abs, cells, or tissues onto a solid-phase carrier can be achieved, resulting in the formation of a dense two-dimensional molecular array. The integration of biochemical analyses on a single chip surface enables swift, precise, and high-throughput detection of target biomolecules. Song et al. [143] designed a paper-based microfluidic chip that incorporates a boronate-affinity metal–organic framework and molecularly imprinted polymer for the swift detection of hazardous veterinary drugs in food. The chip utilizes a uniform zeolite-8 membrane framework as both the support and enrichment layers, employing a highly targeted boronate-affinity surface imprinting technique to develop the recognition layer. The detection of antibiotic residues, such as kanamycin, was accomplished through liquid flow channels, with high specificity and visualization. The entire analysis process is rapid and efficient and can be completed within 30 min. Mi-Sun et al. [144] developed a simple and sensitive method for the detection of enrofloxacin. In this method, a monoclonal Ab specific to enrofloxacin is immobilized on the chip, and the fluorescent microspheres are covalently bound to enrofloxacin molecules. On the basis of the principle of competitive binding between the enrofloxacin in the solution and the enrofloxacin fixed on the microspheres (enrofloxacin-MPs), high dynamic range detection of enrofloxacin was achieved.

#### 6.5.2. Molecular Imprinting Technique

Molecular imprinting technology is a technique that generates MIPs with sizes, shapes, and functional groups that are complementary to those of a template molecule, customizing the binding sites. MIPs possess a specific spatial arrangement, and the polymers not only recognize the size and shape of molecules but also form covalent or noncovalent bonds with the target molecule to attain specific binding capabilities. Molecular imprinting is also referred to as “biosynthetic Abs.” Chemically produced MIPs outperform Abs in terms of specific adsorption of target materials, simplicity in production and preparation, short cycle, low cost, mild storage conditions, and material stability (resistance to high temperature, high pressure, and solvent erosion) and are extensively utilized in environmental monitoring, food analysis applications, and drug analysis. MIPs generally exhibit strong affinity, specificity, and stability and can selectively adsorb specific compounds in complex matrix samples. The application of molecular imprinting in VDR detection mainly involves filling the solid-phase extraction tower with the filler, resulting in the enrichment, purification, and separation of specific compounds. For example, Wen et al. [145] combined MIPs with functional materials such as MOFs and COFs and expanded their application scope in sample processing. Additionally, molecular imprinting technology can be employed as a biosynthetic biosensor in VDR detection, where the analyte is combined with the recognition element to generate a chemical signal that is converted into an electrical or physical signal for molecular measurement. Recently, Zhang et al. [146] synthesized a unique molecularly imprinted CoWO_4_/g-C_3_N_4_ nanomaterial through a combination of hydrothermal and electrochemical polymerization methods. This MIP-CoWO_4_/g-C_3_N_4_ nanocomposite serves as an innovative sensing platform for detecting furazolidone and has a low detection limit of 2.61 × 10^−13^ mol/L and considerable sensitivity.

## 7. Conclusions

The presence of VDRs in animal-derived foods represents a critical and multifaceted challenge to global public health and food safety. Antibiotics, in particular, are the most frequently detected residues, and their misuse and overuse in animal husbandry contribute to a wide range of health risks. Human exposure to these residues occurs primarily through dietary intake, drinking water, and occupational contact, which are pathways that facilitate the entry of these compounds into the human body and subsequently lead to adverse health effects. These effects include acute and chronic toxicities, allergic reactions, disruptions to the gastrointestinal microbiota, and the development of antibiotic resistance. Addressing this challenge demands a holistic mitigation framework. Foremost, the veterinary sector must adopt evidence-based drug stewardship, emphasizing preventive healthcare, precision diagnostics, and optimized therapeutic protocols to minimize antibiotic dependency. Concurrently, regulatory authorities should curb drug abuse through multiple approaches, including policy coordination, enhanced industry oversight and education, economic incentive mechanisms, and the establishment of traceability systems for aquaculture. Complementing these measures, the search for antibiotic alternatives—including bacteriophage therapy, engineered antimicrobial peptides, and plant-based additives—supports more sustainable livestock production by reducing reliance on conventional drugs. In parallel, advances in VDR detection are making food safety monitoring more precise, efficient, and adaptable. While established methods like GC/LC remain essential for analyzing complex samples, and rapid on-site tests like ELISA are still widely used, they are now being effectively combined with non-destructive tools. Real-time spectroscopic techniques, such as Raman and NIR spectroscopy, are key examples. The integration of biosensors with materials like molecularly imprinted polymers has also improved molecular selectivity and testing throughput. Together, this multi-layered strategy will help build more resilient food safety systems, enabling better contamination control and safer food supplies.

In summary, we have gained a complete understanding of the sources, exposure pathways, health impacts, mitigation, and safety assurance of VDRs in the food chain. Thus, the residues of veterinary drugs in the food chain are complex food safety issues that involve livestock practices, veterinary science, public policies, and consumer behavior. Although it is unrealistic to eliminate residues completely, through a sound and effective “farm-to-table” comprehensive management system, including strict regulation, responsible medication, continuous monitoring, and technological innovation, we can effectively control its risk while ensuring animal health and production efficiency and ensuring public food safety and health.

## Figures and Tables

**Table 1:** Tabular Overview of Veterinary Drug MRLs in Major Global Markets [43,44,45,46].

Product Category	Veterinary Drug Class/Name	EU MRL	US MRL	China MRL	Codex MRL	Key Data Sources (Links)
Milk	Penicillin G (β-lactams)	4 µg/kg	ND	4 µg/kg	-	[43,44,45,46]
	Enrofloxacin and ciprofloxacin	100 µg/kg	-	100 µg/kg	-	
	Sulfonamides (total)	100 µg/kg	10 µg/kg	100 µg/kg	25 µg/kg	
Chicken (muscle)	Tetracyclines (total)	100 µg/kg	2000 µg/kg	100 µg/kg	200 µg/kg	
	Gentamicin (Aminoglycosides)	-	100 µg/kg	100 µg/kg	-	
	Chloramphenicol	ND	ND	ND	ND	
Eggs	Enrofloxacin (Fluoroquinolones)	ND	ND	10 µg/kg	-	
	Gentamicin	-	ND	ND	-	
	Tetracyclines (e.g., Chlortetracycline)	200 µg/kg	400 µg/kg	400 µg/kg	400 µg/kg	

**Table 2:** Alternative Strategies to Antibiotics.

Alternative Strategy Against Antibiotics	Mechanism of Action	Key Advantages	Major Limitations	References
probiotics	Competitive exclusion of intestinal pathogens is achieved, alongside the secretion of antimicrobial metabolites, to modulate gut microbiota and enhance barrier function.	High safety profile; promotes growth and improves gut health; cost-effective and easy to administer.	Efficacy is strain-, dose-, and host-dependent; influenced by environmental factors.	[103,104]
phage	Genetic material is injected into host bacteria for specific infection, followed by replication using host cellular machinery, ultimately leading to cell lysis.	High target specificity, sparing commensal flora; self-replicating; effective against ARB; biodegradable with low environmental impact.	Narrow host range; potential for bacterial resistance development; susceptible to gastric acid and immune clearance	[105,106]
Organic Acids	Modulation of gut microbiota and inhibition of pathogenic bacteria by lowering gastrointestinal pH.	Broad antimicrobial spectrum; enhances digestive enzyme activity and nutrient absorption; low risk of resistance; economical.	High doses may reduce palatability; efficacy affected by dietary buffering capacity.	[107,108]
antimicrobial peptide	Primary disruption of bacterial cell membrane integrity, with some variants also exhibiting immunomodulatory properties.	Rapid action; low propensity for resistance development; multifunctional (e.g., immune modulation).	High production cost; susceptible to proteolytic degradation in vivo; challenging to produce at scale.	[109,110]
botanical extracts	Complex actions including disruption of bacterial membranes, inhibition of quorum sensing, and provision of antioxidant and anti--inflammatory effects.	Natural origin; low resistance risk; often multifunctional (antioxidant, anti-inflammatory, immune-supporting).	Variable active ingredient content; potential palatability issues; optimal dosage and mechanisms require further research.	[111,112]
Nanoparticles	Membrane disruption, ROS generation, DNA/protein damage, and metal ion release collectively lead to bacterial cell death.	Multi-mechanistic action reduces resistance risk; can serve as targeted delivery vehicles.	Biosafety concerns (e.g., toxicity, bioaccumulation) are not fully defined; high cost; difficult to scale production.	[113,114]
antibody	Direct targeting of the bacterial surface or indirect neutralization of bacterial toxins and virulence factors that causezation of the bacterial toxins and virulence factors that give rise to infections.	Exceptional specificity and safety; no residue concerns.	High production cost; limited to single targets; oral delivery often inefficient (typically administered via injection or yolk antibodies).	[115,116]

**Table 3:** Analytical methods for veterinary drug residue detection.

Classification	Method	Sensitivity	Cost	Analysis Principle	Examples	References
Chemical analysis method	GC	Ppb level	Medium–High cost. Moderate instrument cost, increases with high-sensitivity detectors (e.g., MS).	The sample components are carried by the carrier gas after vaporization, and the separation action of the chromatography column separates different components. Finally, the detection signal is obtained at the detector.	A column-pretreatment derivatization-GC–MS/MS method for determining decoquinate residues in chicken tissue	[122]
	LC	Ppb level	Medium–High cost. Moderate instrument cost, increases with high-sensitivity detectors (e.g., MS).	The sample solution is introduced into the chromatographic column via a high-pressure pump, where the sample components undergo varying degrees of distribution between the mobile phase and the stationary phase, thereby achieving separation.	UPLC-QTOF-MS technique was employed for the simultaneous quantification of residues of 20 veterinary drugs and their metabolites; PRiME extraction and UHPLC-Q-LIT/electrostatic field orbitrap HRMS were utilized for the detection of various veterinary drugs in sheep milk products.	[123,124]
immunological technique	ELISA	Ppb level	Low–Medium cost. Low instrument cost; costs mainly from kits or antibody development.	Based on the specific binding between antigen and Ab, the separation and detection of target substances are achieved.	A highly sensitive colorimetric biosensor based on nanofiber membrane for on-site detection of chloramphenicol was developed by combining ELISA with nanofiber membrane; utilized the principle of aptamer-based competitive recognition to achieve quantitative detection of chloramphenicol in fish; developed HRP-labeled nanobodies for the detection of enrofloxacin in milk and animal tissues.	[125,126,127]
	CGIA	Ppb-ppm level	Very low cost. Low test strip production cost; no instrument needed.	Using colloidal gold as a tracer label, the reaction is completed during chromatography through the principle of specific antigen-Ab binding, thereby achieving the purpose of detection.	Rapid, on-site, semiquantitative detection of tetracycline in seawater; detection of aldicarb in agricultural products and the environment.	[128,129]
	CLIA	Ppb level	Medium–High cost.	Combining high-sensitivity chemiluminescence detection technology with high-specificity immune reactions, the immune recognition process generates corresponding detectable light signals.	CLEIA for the quantitative measurement of sulfamethoxazole; CLEIA based on HRP-luminol-hydrogen peroxide (H_2_O_2_) chemiluminescence system for the detection of enrofloxacin.	[130,131]
	FPIA	Ppb level	Medium cost.	A known concentration of fluorescently labeled antigen is employed to compete with the target (antigen) for a fixed quantity of Ab. The detection is accomplished by measuring the relationship between the fluorescently labeled antigen and the polarization of the fluorescence.	Detecting amantadine in chicken meat; testing for ractopamine in pork.	[132,133]
	FLISA	Ppb level	Medium cost.	Detection is performed based on the optical properties of quantum dots and the principle of specific antigen-Ab binding.	Precise quantification of florfenicol residues in animal-derived food and feed; Simultaneous, sensitive, and visual detection of streptomycin, tetracycline, and PC-G in milk.	[134,135]
	TRFIA	Ppt-ppb level	High cost. Requires time-resolved fluorescence analyzer.	Lanthanide elements are labeled onto Abs or antigens as tracers. When labeled antigens and Abs form immunocomplexes, the fluorescence intensity of these complexes can be measured using a time-resolved fluorescence analyzer.	The strip test based on TRFMs-LFIA has achieved accurate quantitative and qualitative analysis for both ceftiofur and desfuroylceftiofur.	[136]
spectrum technology	SERS	Ppb level	Medium cost.	Analyzing the scattered light spectrum at different frequencies from the incident light can provide information on the chemical structure, phase, morphology, crystallinity, and molecular interactions of the sample, enabling rapid qualitative and quantitative analysis of different components.	SPE-SERS method is used to detect tetracycline sodium, sulfadiazine, and benzylpenicillin in dairy products; SERS is combined with spectral preprocessing techniques to detect nitrofurantoin in honey.	[137,138]
	NIRS	Ppb-ppm level	Low–Medium cost.	By detecting the absorption or reflection spectrum of a substance in the near-infrared spectral range, chemical composition information of the sample can be obtained.	Detecting antibiotic residues in milk; Determination of Sulfadiazine and sulfamethazine in shrimp paste.	[139,140]
Biosensor technology		Ppt-ppb level	Low–High cost, varies widely.	Using component recognition as the biochemical sensing unit, the appropriate energy conversion principle is used to convert recognizable biological signals into detectable electrical, optical, acoustic, or thermal signals.	An AuNCs-MLFIA sensor based on highly luminescent gold nanoclusters is used to detect clenbuterol and ractopamine; ZnSQDs and PANI nanoparticles are combined with gold electrodes and clenbuterol Abs to form a highly sensitive electrochemical immunoassay sensor for clenbuterol detection.	[141,142]
molecular biological technique	biochip technology	Ppb level	High cost (R&D and manufacturing).	By relying on the specific interactions between biomolecules, a miniature biochemical analysis system can be established, in which biochemical analysis is integrated onto the same chip surface through microfabrication and microelectronics technologies, thereby enabling the detection of target biomolecules.	A paper-based microfluidic chip combining boric acid-affinity MOF and molecular imprinted polymer was designed for high-specificity and visual detection of kanamycin; A monoclonal Ab specific for enrofloxacin was immobilized on the chip based on the principle of competitive binding, enabling high dynamic range detection of enrofloxacin.	[143,144]
	molecular imprinting technique	Ppb level	Medium cost.	MIP has a certain spatial arrangement, and the polymers are not only able to recognize the size and shape of molecules but also able to form covalent or noncovalent bonds with the target molecules to achieve specific binding and detection.	MIPs are combined with functional materials such as MOFs and COFs for VDR detection; molecularly imprinted coWO4/g-C3N4 nanomaterials are used for the detection of furazolidone.	[145,146]

## Data Availability

No new data were created or analyzed in this study. Data sharing is not applicable to this article.

## Abbreviations

The following abbreviations are used in this manuscript:

5-HT	5-hydroxytryptamine
ADI	Acceptable Daily Intake
APIs	Active pharmaceutical ingredients
AGPs	Antibiotic growth promoters
ARGs	Antibiotic resistance genes
ARB	Antibiotic-resistant bacteria
Abs	Antibodies
AMR	Antimicrobial resistance
CLIA	Chemiluminescence immunoassay assay
CGIA	Colloidal gold immunoassay assay
DG SANTE	Directorate-General for Health and Food Safety
ELISA	Enzyme-linked immunosorbent assay
EU	European Union
FLISA	Fluorescence immunoassay assay
FPIA	Fluorescence polarization immunoassay assay
FAO	Food and Agriculture Organization
FDA	Food and Drug Administration
FSIS	Food Safety and Inspection Service
GC	Gas chromatography
GLASS	Global Antimicrobial Resistance and Use Surveillance System
GEMS/Food	Global Environment Monitoring System-Food Contamination Monitoring and Assessment Programme
HRMS	High-resolution mass spectrometry
HGT	Horizontal gene transfer
ICD	Inspection Classification Database
JECFA	Joint FAO/WHO Expert Committee on Food Additives
LC	Liquid chromatography
MRLs	Maximum Residue Limits
MGEs	Mobile genetic elements
MIPs	Molecularly imprinted polymers
MDR	Multidrug resistance
NIRS	Near-infrared spectroscopy
ND	Not Detected
RASFF	Rapid Alert System for Food and Feed
SAMR	State Administration for Market Regulation
SERS	Surface-enhanced Raman spectroscopy
TRFIA	Time-resolved fluoroimmunoassay assay
UNEP	United Nations Environment Programme
US	United States
VDRs	Veterinary drug residues
WHO	World Health Organization
WOAH	World Organization for Animal Health
ZnSQDs	Zinc sulfide quantum dots
